# The Emotional and Ethical Dilemmas of Body Integrity Dysphoria: A Case Report From England

**DOI:** 10.7759/cureus.71121

**Published:** 2024-10-09

**Authors:** Unsa Athar, Louise Johnstone, Chinwendu Udegbe

**Affiliations:** 1 Psychiatry of Intellectual Disability, Mersey Care National Health Service (NHS) Foundation Trust, Liverpool, GBR; 2 Psychology, Mersey Care National Health Service (NHS) Foundation Trust, Liverpool, GBR; 3 General Adult Psychiatry, Mersey Care National Health Service (NHS) Foundation Trust, Liverpool, GBR

**Keywords:** amputation, autism spectrum condition, body integrity dysphoria, icd-11, nhs

## Abstract

Body integrity dysphoria is defined as a rare disorder with the characteristic feature of a persistent desire to have a physical disability, usually specific. Some people with body integrity dysphoria reach a stage where they search for surgical or self-removal of their body part/s.

The aim of presenting this case report is to disseminate information about body integrity dysphoria and the emotional and ethical challenges that haunt the diagnosis.

We present a case of a 52-year-old white British gentleman whom we diagnosed with body integrity dysphoria as he met all the International Classification of Diseases 11th Revision (ICD-11) criteria for diagnosis. Two months before his presentation to us, he went to the train tracks near his residence, also adjacent to a tertiary care hospital, with the intent to have his lower limb amputated by a moving train. He has wished to be an amputee since his childhood. He denied any intent or plan to end his life. He did not wish for an accidental death while trying to be an amputee. All other diagnoses were ruled out. As an infant, he was diagnosed with tetralogy of Fallot and underwent corrective surgery. A year ago, he was referred to secondary mental health services via his general physician and eventually received a diagnosis of autism spectrum condition. The patient himself has theorised that perhaps his ‘underdeveloped heart led to poor oxygenation to his brain, or maybe his brain was broken in some way, which causes the heart’s misconfiguration’. His management on the ward involved work with psychology. Rather than trying to 'change' the patient or the nature of his thoughts, as this would likely cause more distress and non-engagement, the psychological treatment plan was to focus on reducing risk.

Many speculations have been made regarding the aetiology. However, the therapeutic difficulties remain a challenge in 2024 as the disorder is understudied. A literature review published in 2021 suggested that amputation remains the most ‘satisfying’ management strategy, even though it is shunned in the medical community. A once misunderstood and misdiagnosed disorder, body integrity dysphoria has been appreciated by the ICD-11 as a separate entity. Our duty of care urges us to understand not just the biology of the ailment but also the ethically questionable resorts used so far (self-amputation) to deal with the emotional turmoil of wanting to be an amputee. A holistic and personalised biopsychosocial model of care is needed for patients with body integrity dysphoria.

## Introduction

The World Health Organization regularly works on modifying and improving the International Classification of Diseases (ICD) and Related Health Problems. The 11th revision of ICD (ICD-11) was approved by the World Health Assembly in 2019 to take effect from January 2022 [1]. ICD-11 introduced a new category named ‘disorders of bodily distress and bodily experience’, which includes two subcategories - bodily distress disorder and body integrity dysphoria. Bodily distress disorder encompasses somatoform disorders present in ICD-10. Body integrity dysphoria is defined as a rare disorder with the characteristic feature of a persistent desire to have a physical disability, usually specific. The desire includes fantasising about it, pretending to be a disabled person and spending an adequate amount of time planning how to achieve this wish. These activities impact all areas of the person’s life, professionally and personally. Some people with body integrity dysphoria reach a stage where they search for surgical or self-removal of their body part/s [1].

Body integrity dysphoria is coded as 6C21 [2]. It was previously known as body integrity identity disorder (BIID), along with other names [3]. Reports from Scotland came through in 2000 where surgeons amputated healthy legs for individuals who struggled with their abled-bodied identity [4]. With increasing reports in the literature, emphasis was given to including this disorder as a discrete identity [5]. Calculating the true incidence and prevalence of this condition is a practical challenge as people do not come to the direct attention of healthcare providers; most of them never share their thoughts with other people owing to stigma. However, it has been suggested that the disorder might not be as rare as we thought [6]. Body integrity dysphoria poses a therapeutic challenge to healthcare professionals. Selective serotonin reuptake inhibitors are used to manage the distress that comes from not being able to achieve the disability [6]. Psychotherapy appears to be the mainstay of management, with a German article mentioning a ‘successful’ two and half years of psychotherapy for a gentleman and suggesting that cognitive behavioural therapy integrated with psychodynamic therapy might be needed to form a treatment approach [7].

We present to you a case of a 52-year-old white British gentleman, called Mr. A, for the sake of this write-up, who was diagnosed with body integrity dysphoria while in our care. The aim of presenting this case is to highlight the emotional and ethical turmoil of suffering from and managing such a complex condition.

## Case presentation

Presenting illness

Mr. A was transferred to a National Health Service (NHS) General Adult Psychiatry ward in Liverpool, England, from a Private General Adult Ward in Leicester. Two months before his presentation to us, in September 2023, he went to the train tracks near his residence, also adjacent to a tertiary care hospital, with the intent to have his lower limb amputated by a moving train.

History of presenting illness

Before his decision to go to the rail tracks, he had eight cans of beer, which he described as ‘liquid courage’. He was then saved by the police present at the railway station. He was escorted back to his home, where he tried to cut his leg off with a saw machine. He was then taken to the emergency department under section 136 of the Mental Health Act (MHA). Later, he was sectioned using section 2 of the MHA 1983 (which was his first admission via MHA). Due to the lack of beds in the NHS, he was temporarily shifted to a private healthcare facility until an NHS bed was found in his area. During his first ward interview, he expressed that he has been having thoughts of being an amputee ‘for as long as I can remember’. He was fully aware of the consequences of being an amputee. ‘I feel like I was born in the wrong body. I should have been in the body of an amputee’. His first recall of this desire was when he was in pre-school. He remembers seeing a girl with a bandage around her knee as she recently had a below-knee amputation, and he thought to himself, ‘Oh, this is the coolest thing I have ever seen.' Following that thought, his desire to be an amputee has always stayed there. The thoughts were more intense during the ‘happy’ periods of his life, but he would be able to cope with them. However, when ‘life stressors get the best of me’, he makes attempts to achieve his ‘dream body’.

He has made multiple attempts to lose his limbs. At work, he would see a ‘large wood-cutting machine and think, "Oh wow, I could make it look like an accident; I could lose a limb here”’. He has tried to burn his fingers off, tourniquet his limbs, and insert paint into his foot that might lead to the blockage of blood flow into the toes, and he would lose them eventually. He studied prosthetics and crutches extensively. There have been occasions where he would wait for his family to leave home and then strap a prosthetic leg to his limb and walk around the house as an amputee. He would also use crutches around the house. He bought these devices online to live his dream as a reality in some moments. He did not wish to die. He never had any thoughts, ideations, or intent to end his life. All his attempts to lose his limb involved safe procedures, not life-threatening. He wished for a ‘clean cut’ and did not wish to ‘bleed out’. Many years ago, he did not remember exactly when he even went to a surgeon to request an amputation but was turned away as it was ‘unethical’.

Personal and developmental history

Divulging further into his life, he told the authors that he was born via spontaneous vaginal delivery. He has an elder brother. When he was 18 months old, he was diagnosed with tetralogy of Fallot because he was ‘passing out a lot’. In 1972, he underwent a surgical correction for the procedure, and ever since, he has been having annual reviews with cardiology. He grew up in a ‘healthy household’. He expressed that his parents took ‘extra care of me, maybe then went overboard because they saw me struggling in life’. He reached all developmental milestones in a timely. In school, he remembers being bullied for having ‘man boobs because of the horizontal chest scar after the surgery’, but ‘I dealt with it by ganging up with the bullies and finding other kids we could pick on’. He made friends in school that he kept as his friends throughout his adulthood.

Social history

After his General Certificate of Secondary Education (GCSE), he started working at a timber yard and has worked there to date. He would sometimes struggle with money, as the job is not well paid, but he described his parents as ‘always being there to help me with mortgage and other finances’. His parents are in their 70s now and settled in the same city. His brother is married and settled in another county. He remembers having a great relationship with him. Throughout his life, he struggled with the thoughts and intense desire to be differently abled. 

Journey with autism

In 2022, he met the ‘love of my life', B, and decided to marry her. The ‘pressure’ of his life changing led him to ‘having a breakdown at work’. He expressed that he started bawling at work when he failed to amputate himself; the attempt to amputate was not witnessed by his work colleagues at the time, and thus, they were not aware he felt this way. He was struggling with low mood associated with social difficulties. After taking a detailed history, his general physician (GP) referred him to secondary mental health services for Autism Assessment in August 2022. In the same year, he eventually received a diagnosis of autism spectrum condition (ASC), with difficulties identified in the following domains: social communication, social interaction and social imagination. He framed the diagnosis as ‘answering some questions but raising more questions for me about my life’. After receiving the diagnosis, he eventually ‘came to terms with some aspects of my mental health’. His GP initiated him on citalopram 20 mg in October 2022 due to repeated reports of low mood.

Impact of alcohol

He denied any recreational drug use but had episodes of 'binge drinking', drinking up to ‘eight pints of 4% lager’ to ‘give him enough courage to act on the thoughts’. He did not believe he had an issue with alcohol. On further exploration, it was reported that he would binge drink eight pints of 4% lager every Friday, Saturday and Sunday (each pint of 4% Lager is 2.3 units) and a total of eight pints on Monday to Thursday. He has followed this pattern of drinking every month since 2022. His adds up to a total of 73.6 units weekly. He was referred to the Inpatient Addictions Team. He engaged well with them and requested a referral for alcohol support services in the community.

Relationship with wife

He lived with his wife, B, without other family members. He does not have any kids. However, she has a 29-year-old son from a previous partner and a grandson. He says that he ‘loves my wife to bits’, but since the marriage, he has not been able to ‘shake off’ the thoughts of losing a limb. He kept his crutches, prosthetic limbs and saw at home, hidden from her. B worked intermittent night shifts for a month, and that was the time he used to use his prosthetic limbs and crutches around the house or act on amputating himself. He once burnt his foot trying to ‘get rid of it’, but when asked by her, he lied that he had dropped a kettle on it. He opened up to his wife during the current admission. His wife has been supportive since then. He said that she ‘would accept me even if I was amputated as she loves me’.

Ruling out other diagnoses

He denied feeling like his body or body parts were ‘ugly or deformed’. He felt congruent with his gender assigned at birth. He felt pain and other sensations equally in all limbs. He denied having a hallucinatory or delusional experience that made him feel like his limbs did not belong to him or were ‘alien.’ He did not report or express further perceptual or thought deformities. He expresses distress over ‘not being an amputee/differently abled’ but not from having these thoughts. He did not believe them to be intrusive. Nor did he wish to seek care from health professionals or secondary gains, as he never shared these thoughts with anyone to achieve such benefits. Thus, malingering was ruled out, as he never acted upon these thoughts in the context of secondary gains. ‘I do not do this for show. I truly want to become an amputee’. He did report ‘sometimes being turned on by disabled people or by thoughts of him being amputated’, but it was on rare occasions and not the primary focus of these thoughts.

Progress on the ward

He was admitted under the MHA 1983 to a private ward in September 2023. His citalopram 20 mg was continued, and he was also initiated on that ward. He was transferred to our ward in October 2023. The rationale behind the use of aripiprazole was queried, and it was discontinued as he reported no benefit from it. He wished to continue his anti-depressant as it took ‘the edge off the distress of not being an amputee’. His citalopram was changed to fluoxetine 20 mg, and the aripiprazole was discontinued. During his admission, he attempted to tie his limbs in the initial days with a shoelace but was encouraged by staff to open up the knot and hand over the lace. During this admission, he would hand over multiple items to staff, ranging from trousers to ties. He would not open up initially on why he was handing these over, but then later in psychology, he admitted that he did not want to act on the thoughts and that the items he handed over were the ones he could potentially use. He engaged well in psychology, the formulation of which is mentioned later. During the course of two weeks, he felt he was safe enough to go home. His thoughts involving amputation were still there, but he felt better equipped to manage those. He was discharged in the first week of November 2023. Safe discharge planning involved referring him to the Community Mental Health Team (CMHT) for care coordination. A referral to psychological services in the community could then be explored. He was discharged on fluoxetine 20 mg once a day.

Physical health

After the surgical repair of the tetralogy of Fallot in 1972, he was put on bisoprolol 1.25 mg once a day. He was diagnosed with gout in 2005. He has been trialled on celecoxib 200 mg capsules, colchicine 500 μg tablets and febuxostat 80 mg to poor effect. He was initiated on allopurinol in August 2024.

Upon admission, a full blood count, liver function tests, bone profile, renal function tests, thyroid function tests, glycated haemoglobin (HBA1c), vitamin B12, folate, prolactin and serum lipid profile were ordered. His serum cholesterol to high-density lipid ratio was elevated (8), and he wished to try lifestyle modifications to deal with that.

His understanding of the condition

When he asked if he ever thought about why he might have those thoughts, he expressed that he has mulled over it a lot. In his mind, he has theorised that perhaps his underdeveloped heart led to poor oxygenation to his brain, or maybe his brain was broken in some way, which caused the heart’s misconfiguration. Last but not least, he consented to us to present his story for learning for our colleagues. He shared with us that he has been going to a cardiologist every year and never shied away from doctors or students who wished to listen to his murmurs or look at his scar. ‘I live with the knowledge that I might drop dead any minute. Why not contribute to science while I am living?’

Psychological formulation

The consultant psychologist on the ward had one session with him to help with the formulation. The psychologist believed that the different sensory needs of neurodivergent people might contribute to his presentation. Her management plan for the patient was rather than trying to 'change' the patient or the nature of his thoughts, as this would likely cause more distress and non-engagement, the psychological treatment plan was to focus on reducing risk. This involved supporting the patient to engage in low-risk behaviour and teaching them skills to cope with anxiety and to manage the distress that came with feeling in the ‘wrong body’.

Follow-up

After his discharge from the ward, he was seen by the community General Adult Psychiatrist, who pinned his primary issues to be his alcohol use and his anxiety associated with autism/ borderline personality disorder (BPD). His fluoxetine was increased to 40 mg once a day, which he has continued to this day. He also started engaging with Change Grow Live (CGL) Integrated Recovery Service to address his alcohol use. He has been allocated a keyworker, and with their help, he has cut down his alcohol intake to three cans of 4% lager on Friday and Saturday. There are some weeks where he only drinks one day per week. As his engagement with CGL has been positive in stabilising his mental health, he was discharged from the CMHT in February 2024 with a request for referral to the CGL Psychology team. 

## Discussion

The description above exhibits that our patient, A, fulfils the criteria for body integrity dysphoria as characterised by the ICD-11. Many speculations have been made regarding the aetiology. Neurological imaging done on patients with this disorder has theorised abnormalities in the ‘frontoparietal/body ownership network,’ an increase of grey matter in the cerebellum and an increase in left dorsal and ventral premotor cortices [8]. Additionally, links to social circumstances have also been found [9]. The therapeutic difficulties remain a challenge in 2023, as the disorder is understudied. A literature review published in 2021 suggested that amputation remains the most ‘satisfying’ management strategy, even though it is shunned in the medical community [9]. Looking at the case presentation of a 24-year-old transgender male published in 2022 [10], the authors were forced to query the role of amputation and the ethical questions it raised. This gentleman (assigned female at birth) also struggled with gaming disorder, schizoid personality traits and bipolar affective disorder. He ended up amputating his left hand in a safe manner after meticulous planning. He left a handwritten note requesting medical help to save his life if need be but used caustic agents before sawing this hand to prevent surgical correction of his attempt. This gentleman also used the support of ‘online peer communities’ while performing the self-amputation.

Online websites and communities were explored in 2018 [6], and it was brought to light that these communities are essentially made as peer support for people who have the desire to become disabled. People often also share their ‘success stories’ of self and surgical amputations. This leads to them being vulnerable to financial and emotional exploitation, luring them in with offers to amputate in a ‘safe and healthy manner’ for financial gains. Ethical considerations were questioned in 2009 by a paper that queried if this is truly a neurological disorder and whether the patients have enough insight to make a capacious decision to consent to amputation [11]. This was further analysed by Barrow and Oyebode [5], who also discussed in their article the impact on the economy vis-à-vis the economic burden of providing facilities to an increasing number of ‘self-created’ disabled people. However, Barrow and Oyebode concluded that despite our discomfort with the thoughts the patients have, they deserve our compassion and complete duty of care. This duty of care thus must encourage us to find safer, ‘more ethical’ alternatives to management. Vestibular caloric stimulation is under speculation but was not found effective in earlier studies [9]. An article published in 2020 suggests using neurofeedback mechanisms with a computer interface that have previously been successful in managing post-traumatic stress disorder [12]. It has been theorised that using transcranial magnetic stimulation (TNF) and studying the neurofeedback motor evoked potentials might help us target the brain areas affected by body integrity dysphoria [13], which might become potentially beneficial. Another novel technique was employed in Amsterdam. Augmented reality telepresence to the ideal self (ART-IS) was used in two patients, who reported it to be ‘a positive and welcoming intervention’. The authors of the article acknowledged the limitations of the study [14] but urged scientists to look into augmented reality as a mainstay treatment or an adjunct to neuromodulation or behavioural therapy.

The aim of this case presentation is, as once discussed in correspondence to the British Journal of Psychiatry, to emphasise the importance of case reports [15]. A once misunderstood and misdiagnosed disorder has been appreciated by the ICD-11 as a separate entity. While it is essential to understand the biology behind such distressing ailments, there is a pressing need to find holistic management for the sufferers. Our safeguarding duty of care urges us to present these cases not just as statistics but as human beings with lives, relationships and emotions. Understanding the emotional and ethical turmoil such patients face might tickle our scientific curiosity. In addition, as with all other diagnosable mental illnesses, it is pertinent to develop biopsychosocial management, even if that challenges our own ideas, beliefs and triggers about 'ethically' safe practices.

## Conclusions

A once misunderstood and misdiagnosed disorder, body integrity dysphoria has been appreciated by the ICD-11 as a separate entity. Our duty of care urges us to understand not just the biology of the ailment but also the ethically questionable resorts used so far (self-amputation) to deal with the emotional turmoil of wanting to be an amputee. A holistic and personalised biopsychosocial model of care is needed for patients with body integrity dysphoria.
